# Patterns of failure and long-term outcome of postoperative radiotherapy on the survival of patients with pathological T3N0M0 esophageal cancer

**DOI:** 10.3389/fsurg.2022.959568

**Published:** 2022-09-02

**Authors:** Chunyang Song, Shuchai Zhu, Jinrui Xu, Jingwei Su, Xueyuan Zhang, Wenzhao Deng, Xiaohan Zhao, Wenbin Shen

**Affiliations:** Department of Radiation Oncology, The Fourth Hospital of Hebei Medical University, Shijiazhuang, China

**Keywords:** esophageal carcinoma, surgery, postoperative radiotherapy, patterns of failure, prognosis

## Abstract

**Purpose:**

The prognostic effect of postoperative radiotherapy (PORT) on pathological T3N0M0 (pT3N0M0) esophageal squamous cell carcinoma (ESCC) remains inconclusive. This study aimed to retrospectively investigate patterns of failure and whether PORT after R0 resection improves survival in patients with pT3N0M0 ESCC, compared with surgery alone.

**Patients and methods:**

The clinical data of 256 patients with pT3N0M0 ESCC from January 2007 to December 2010 were retrospectively reviewed. The included patients were classified into two groups: the surgery-plus-postoperative radiotherapy group (S + R) and the surgery-alone group (S). Propensity score matching (PSM) was used to create comparable groups that were balanced across several covariates (*n* = 71 in each group). Statistical analyses were performed using the Kaplan–Meier method and Chi-squared test.

**Results:**

In the study cohort, the 5- and 10-year overall survival (OS) rates in the S + R group were 53.4% and 38.4%, and those in the S group were 50.3%, 40.9% (*p* = 0.810), respectively. The 5- and 10-year disease-free survival (DFS) rates in the S + R group were 47.9% and 32.9%, and those in the S group were 43.2%, 24.0% (*p* = 0.056), respectively. The results were coincident in the matched samples (*p* = 0.883, 0.081) after PSM. Subgroup analysis showed that patients with upper thoracic lesions in the S + R group had significantly higher OS than patients in the S group (*p* = 0.013), in addition, patients with upper and middle thoracic lesions in the S + R group had significantly higher DFS than patients in the S group (*p* = 0.018, 0.049). The results were also confirmed in the matched samples after PSM. The locoregional recurrence between the two groups were significantly different before and after PSM (*p* = 0.009, 0.002). The locoregional control rate (LCR) in the S + R group was significantly higher than that in the S group before and after PSM (*p* = 0.015, 0.008).

**Conclusion:**

Postoperative radiotherapy may be associated with a survival benefit for patients with pT3N0M0 upper thoracic ESCC. A multicenter, randomized phase III clinical trial is required to confirm the results of this study.

## Introduction

Esophageal cancer (EC) is one of the most aggressive malignant tumors. Among all cancers, EC has the seventh- and fifth-highest morbidity and mortality rates, respectively (1). In China, EC ranks as the fourth most common cause of cancer-related deaths; more than 90% of EC is pathologically diagnosed as esophageal squamous cell carcinoma (ESCC) (2). EC is associated with poor prognosis, with a 5-year overall survival rate not exceeding 30% (2–4). Surgery is still a fundamental therapeutic strategy for patients with EC, according to the Clinical Practice Guidelines in Oncology of the National Comprehensive Cancer Network (NCCN). Neoadjuvant chemoradiotherapy, followed by surgery, is the standard treatment for patients with locally advanced ESCC (5). Nonetheless, surgery as a primary treatment is more acceptable by a number of patients because of the traditional concept in China, although neoadjuvant therapy is recommended by surgeons (6). Locoregional recurrence or distant metastasis remain as main patterns of failure despite the presence of radical resection and extended lymph node dissection (7).

Surgery is the predominant treatment for patients with pathological T3N0M0 (pT3N0M0) ESCC (8), particularly those who are clinically understaged before surgery and receiving no neoadjuvant chemoradiotherapy as primary treatment. Previous studies have shown that pT3N0M0 ESCC after radical resection plus lymphadenectomy have a 5-year overall survival of only 32.4%–58.5%; however, the locoregional recurrence rate reaches 25.0%–42.0% (8–17), suggesting the need to direct increased attention to this subgroup. Therefore, surgery itself is not a sufficient definitive treatment for these patients. Some literature reviews have proved that postoperative radiotherapy (PORT) was beneficial to ESCC patients with stage III or positive lymph nodes (14, 18–22). However, no convincing evidence from prospective studies has thus far been reported that PORT accepted by patients with pT3N0M0 ESCC provides a statistically significant survival benefit. This study aimed to investigate the patterns of failure and to determine whether relative to resection alone, PORT improves survival in patients with pT3N0M0 ESCC after radical resection.

## Methods

All procedures in this study involving human participants were conducted in accordance with the Declaration of Helsinki (as revised in 2013). This study was reviewed and approved by the Ethics Committee of The Fourth Hospital of Hebei Medical University.

### Patient selection

**Figure 1:**
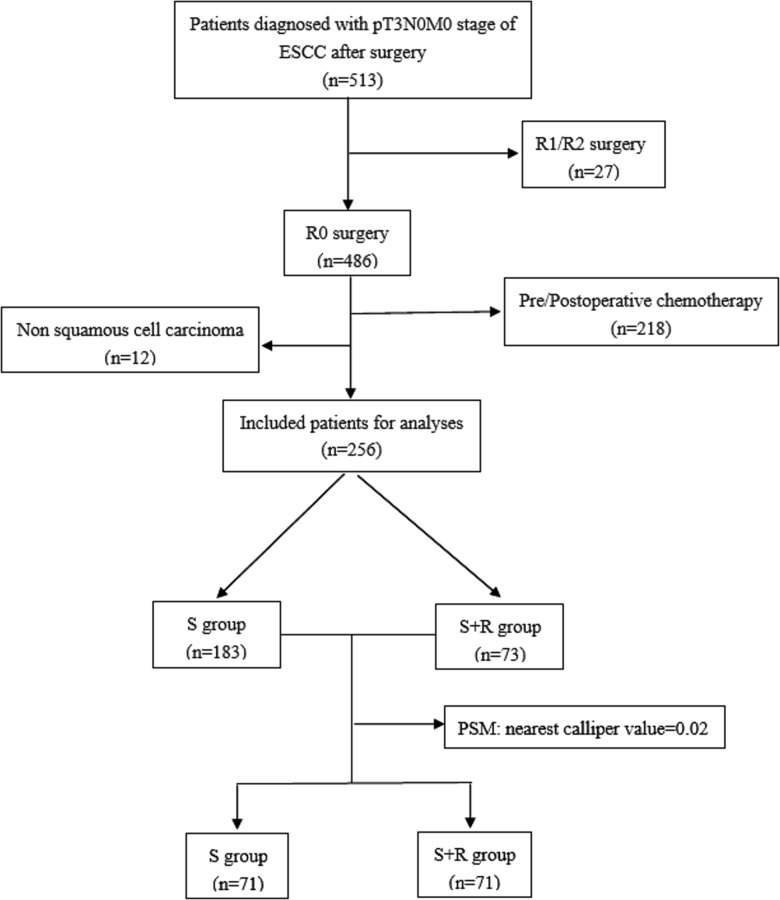
The study flow chart. (ESCC, Esophageal squamous cell carcinoma; S, surgery alone; S + R, surgery plus postoperative radiotherapy; PSM, propensity score matching).

The clinical data of 256 patients with ESCC treated at the Fourth Hospital of Hebei Medical University from January 2007 to December 2010 were retrospectively reviewed for this study. The inclusion criteria were as follows: (1) pT3N0M0 ESCC confirmed in accordance with the Union for International Cancer Control 2009 staging; (2) R0 with esophagectomy plus lymphadenectomy; and (3) the Karnofsky Performance Status (KPS) score ≥80. The exclusion criteria were as follows: (1) non squamous cell histology or multiple primary cancers; (2) perioperative death; (3) absence of required data; and (4) patients who had received neoadjuvant therapy and/or postoperative chemotherapy. The included patients were divided into two groups: the surgery plus postoperative radiotherapy group (S + R) and the surgery alone group (S) (Figure 1).

### Surgery

All enrolled patients were treated with radical esophagectomy. The surgical procedure was determined by the tumor location and preference of surgeon. The most common procedure for middle and lower thoracic esophageal lesions was left thoracotomy, whereas that for upper thoracic esophageal lesions was right thoracotomy. In our study cohort, the number of patients receiving each listed procedure was as follows: left thoracotomy, 220; right thoracotomy, 7; double incision of the neck and upper abdomen, 8; and three incisions of the neck, right thorax, and upper abdomen, 21. Radical surgical resection consisted of esophagectomy and lymphadenectomy in the abdomen and mediastinum. The number of lymph nodes harvested per case ranged from 3 to 28 (median 9). The resected esophagus was replaced with an intrathoracic gastric tube to restore the continuity of the alimentary tract. The anastomotic site depended on tumor location. Upper or middle thoracic esophageal lesions are generally anastomosed in the neck or above the aortic arch. Lower thoracic esophageal lesions were anastomosed above or below the aortic arch. In our study cohort, cervical anastomosis, supra-aortic arch anastomosis, and sub-aortic arch anastomosis were performed in 53, 184 and 19 patients, respectively.

### Postoperative radiotherapy

Radiotherapy was conducted 4–10 weeks after surgery. Digitized images from computed tomography (CT) scan simulation were transmitted to the treatment planning system (ADAC Pinnacle3 8.0 m, Philips Medical Systems). The clinical target volume (CTV) was defined according to the different tumor sites, including the primary site, anastomotic stomas, and the corresponding lymphatic drainage area (which was delineated based on the LN grouping criteria set by the American Thoracic Society). For the patients with upper thoracic ESCC, the CTV borders were defined superiorly as the cricothyroid membrane and inferiorly as 2 cm–3 cm below the trachea carina, including the lower cervical and supraclavicular regions and the mediastinal stations 2, 3p, 4, 5, and 7 and part of 8. For the patients with middle thoracic ESCC, the CTV involved the mediastinal stations 2, 3p, 4, 5, 7, and 8 and the paracardial region. For the patients with lower thoracic ESCC, the CTV involved mediastinal stations 3p, 4, 5, 7, and 8; the paracardial region; the left gastric region; and the celiac trunk region (9). The planning target volume (PTV) was determined by expanding the CTV by 0.5 cm in three-dimensional directions. (The target delineation pictures of CTV with different tumor sites were added as Supplementary Figure S1). A total dose of 50.4 Gy (range, 45–54 Gy; 1.8–2.0 Gy/fraction and 5 fractions per week) using 6-MV photon beams from a linear accelerator was prescribed to 95% of the PTV. Organs at risk (OAR) (9), including the bilateral lungs, spinal cord, gastric tube, and heart, were delineated. The dose to the OAR was limited as follows: a maximal dose to the spinal cord less than 45 Gy; the percentage of irradiated bilateral lung volume exceeding 5 Gy equal to or less than 55%; the percentage of irradiated bilateral lung volume exceeding 20 Gy equal to or less than 28%; the percentage of the irradiated bilateral lung volume exceeding 30 Gy equal to or less than 18%; the percentage of irradiated heart volume exceeding 30 Gy less than 40%; the percentage of irradiated heart volume exceeding 40G y less than 30%; the percentage of irradiated stomach volume exceeding 40 Gy equal to or less than 40%; and the absence of hotspots on the gastric tube.

### Follow-up

Acute and late toxicities were graded based on the Common Toxicity Criteria for Adverse Events, version 3.0. All patients were assessed weekly during treatment and followed up every 3–6 months in the first 2 years after treatment, every 6–12 months in the next 3 years, and annually thereafter. Assessments included a CT scan with contrast of the neck, thorax, and upper abdomen; bone-emission CT; and conventional blood and biochemistry studies. Gastroscopy or positron emission tomography–CT was performed as needed. Treatment failures were classified as locoregional recurrence or distant metastases. Locoregional recurrences were defined as recurrences at the supraclavicular, mediastinal, anastomotic, and upper abdominal (left gastric, celiac trunk) regions. Distant metastases were defined as recurrences at other sites. Overall survival (OS) was measured from the date of surgery to the date of death or last follow-up and censored at the last contact date in surviving patients. Disease-free survival (DFS) was measured either (i) from the date of surgery to the date of the first evidence of relapse or (ii) death from any cause, whichever was observed first. For patients who had not relapsed or died, DFS was censored at the last follow-up date. In this study, the last date of follow-up was August 20, 2020. A total of 256 patients were followed up, and 5 patients were lost to follow-up because of loss of contact. A total of 152 patients died during follow-up, including 70 with locoregional recurrence, 41 with distant metastasis, and 41 with locoregional recurrence and distant metastasis.

### Statistical analyses

Statistical analyses were conducted using the software SPSS 22.0 (IBM Corp, Armonk, NY). Categorical and continuous variables were compared using the Chi-square test and Student t-test or the Wilcoxon rank-sum test. The Kaplan–Meier method was adopted to calculate the survival rate, and the log-rank method was used to compare survival curves between groups. To balance observable potential confounders, PSM analyses were used to create two comparable groups of patients: the S + R group and the S group. We performed PSM separately within each tumor location stratum primarily because surgical resections are expectedly less variable within each tumor location stratum. Therefore, postoperative complications are more likely to be homogenous. Independent variables were entered into the propensity model, including sex, age, history of smoking, preoperative diet, tumor location, length of lesions, differentiation of pathology, number of lymph nodes resected, and degree of adhesion during surgery. Nearest-neighbor matching within a prespecified caliper width without replacement was used as the matching algorithm to perform 1:1 matching of patients in the S + R group and the S group. The significance level was set to *p* < 0.05.

## Results

### Clinical characteristics of the patients

A total of 256 patients—73 in the S + R group and 183 in the S group—met the inclusion criteria and participated in this study. In the S + R group, 29 patients received IMRT, and 44 patients received 3D-CRT. The propensity score-matched cohort included 71 patients in the S + R group and 71 patients in the S group. Good balance was achieved in the covariables of the two groups. The characteristics of the study population are summarized in Table 1.

**Table 1:** Characteristics of study populations.

Characteristic	Before PSM (*n* = 256)			After PSM (*n* = 142)		
	S + R group	S group	*p*-value	S + R group	S group	*p*-value
	*n* = 73 (%)	*n* = 183 (%)		*n* = 71 (%)	*n* = 71 (%)	
Age (year)			0.003			1.000
<62	46 (63.0)	78 (42.6)		40 (56.3)	40 (56.3)	
≥62	27 (37.0)	105 (57.4)		31 (43.7)	31 (43.7)	
Gender			0.223			1.000
Male	54 (74.0)	121 (66.1)		53 (74.6)	53 (74.6)	
Female	19 (26.0)	62 (33.9)		18 (25.4)	18 (25.4)	
Smoking			0.594			1.000
Yes	36 (49.3)	97 (53.0)		37 (52.1)	37 (52.1)	
No	37 (50.7)	86 (47.0)		34 (47.9)	34 (47.9)	
Preoperative diet			0.870			0.212
General food	53 (72.6)	131 (71.6)		51 (71.8)	44 (62.0)	
Half-fluid/fluid food	20 (27.4)	52 (28.4)		20 (28.2)	27 (38.0)	
Tumor Location			0.023			1.000
Upper	20 (27.4)	25 (13.7)		18 (25.4)	18 (25.4)	
Middle	34 (46.6)	112 (61.2)		34 (47.9)	34 (47.9)	
Lower	19 (26.0)	46 (25.1)		19 (26.8)	19 (26.8)	
Length of lesions (cm)			0.694			0.593
<5	26 (35.6)	70 (38.3)		25 (35.2)	22 (31.0)	
≥5	47 (64.4)	113 (61.7)		46 (64.8)	49 (69.0)	
Differentiation of pathology			0.621			0.831
Moderate or well	155 (84.7)	60 (82.2)		58 (81.7)	57 (80.3)	
Poor	28 (15.3)	13 (17.8)		13 (18.3)	14 (19.7)	
Lymphadenectomy			0.103			0.196
2-field	61 (83.6)	166 (90.7)		60 (84.5)	65 (91.5)	
3-field	12 (14.4)	17 (9.3)		11 (15.5)	6 (8.5)	
Surgery procedure			0.059			0.098
Left-sided	58 (79.5)	162 (88.5)		57 (80.3)	64 (90.1)	
Right-sided	15 (20.5)	21 (11.5)		14 (19.7)	7 (9.9)	
Numbers of dissected lymph node			0.707			0.397
<9	35 (47.9)	83 (45.4)		33 (46.5)	28 (39.4)	
≥9	38 (52.1)	100 (54.6)		38 (53.5)	43 (60.6)	
Degree of adhesion during surgery			0.255			0.944
No adhesions	1 (1.4)	7 (3.8)		1 (1.4)	1 (1.4)	
Mild adhesions	34 (46.5)	99 (54.1)		33 (46.5)	35 (49.3)	
Severe adhesions	38 (52.1)	77 (42.1)		37 (52.1)	35 (49.3)	

PSM, propensity score matching; S, surgery alone; S + R, surgery plus postoperative radiotherapy.

### Survival

**Figure 2:**
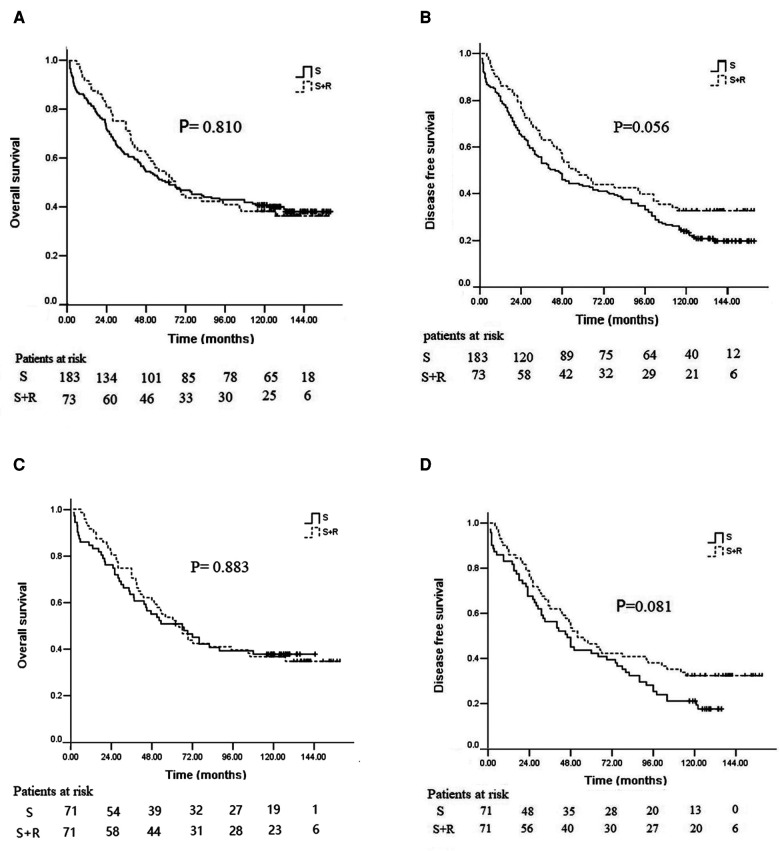
Kaplan–Meier analysis of survival between the S group and the S + R group of ESCC patients with pT3N0M0 before and after PSM. (**A**) OS before PSM; (**B**) DFS before PSM; (**C**) OS after PSM; (**D**) DFS after PSM. PSM, propensity score matching; S, surgery alone; S + R, surgery plus postoperative radiotherapy; ESCC, Esophageal squamous cell carcinoma; OS, overall survival; DFS, disease free survival).

The median follow-up time across the whole study population was 62.4 months (range 1.5–160.3 months). In the overall study cohort, the 3-, 5-, 10-year OS rates were 64.5%, 51.2%, and 40.2%, respectively, and the median OS was 63.0 months. The 5- and 10-year OS rates in the S + R group were 53.4% and 38.4%, respectively; those in the S group were 50.3% and 40.9%, respectively. No significant difference was found between the two groups (*p* = 0.810, Figure 2A). The 3-, 5-, and 10-year DFS rates were 55.9%, 44.5%, and 26.5%, respectively; the median DFS was 48.4 months. The 5- and 10-year DFS rates in the S + R group were 47.9% and 32.9%, respectively; those in the S group were 43.2% and 24.0%, respectively. No significant difference was found between the two groups (*p* = 0.056, Figure 2B).

After PSM, the 5- and 10-year OS rates in the S + R group were 52.1% and 36.6%, and those in the S group were 50.7% and 37.7% in the well-balanced pairs of patients, respectively (*p* = 0.883, Figure 2C). The 5- and 10-year DFS rates in the S + R group were 46.5% and 32.4%; those in the S group were 42.3% and 21.1%, respectively (*p* = 0.081, Figure 2D).

### Subgroup analysis

**Figure 3:**
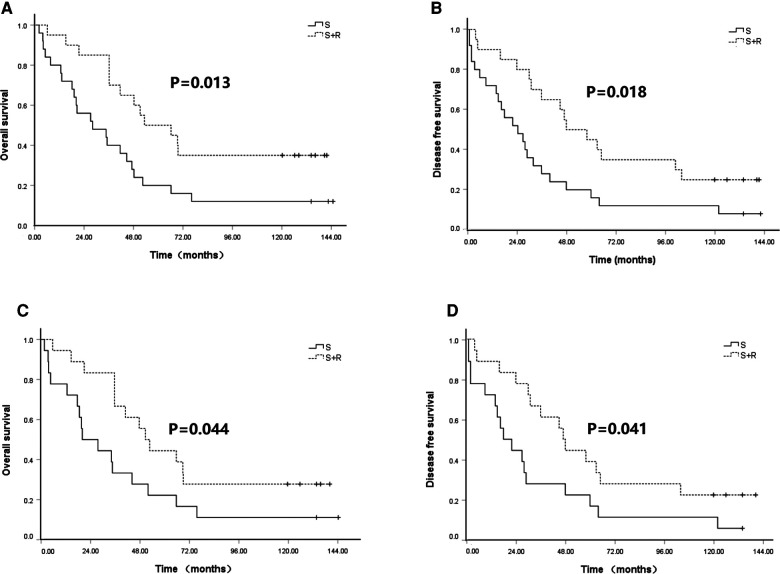
Kaplan–Meier analysis of survival between S group and S + R group for upper thoracic lesions ESCC patients with pT3N0M0 before and after PSM. (**A**) OS before PSM; (**B**) DFS before PSM; (**C**) OS after PSM; (**D**) DFS after PSM. PSM, propensity score matching; S, surgery alone; S + R, surgery plus postoperative radiotherapy; ESCC, Esophageal squamous cell carcinoma; OS, overall survival; DFS, disease free survival).

To identify patients who would benefit from PORT, we performed a subgroup analysis of OS and DFS. We found that patients with upper thoracic lesions in the S + R group had significantly higher OS than patients in the S group (5-year OS of 50.0% vs. 20.0% and 10-year OS of 35.0% vs. 12.0%, *χ*^2^ = 6.233, *p* = 0.013; Figure 3A). No significant difference in OS was indicated between the two groups of patients with middle and lower thoracic lesions (*p* = 0.858 and 0.627). The patients with upper and middle thoracic lesions in the S + R group had a significantly higher DFS than the patients in the S group (upper thoracic lesions 5-year DFS of 45.0% vs. 16.0% and 10-year DFS of 25.0% vs. 12.0%, *χ*^2^ = 5.592, *p* = 0.018, Figure 3B; middle thoracic lesions 5-year DFS of 47.1% vs. 42.0% and 10-year DFS of 32.4% vs. 17.9%, *χ*^2^ = 3.864, *p* = 0.049). No significant difference in DFS was found between the two groups of patients with lower thoracic lesions (*p* = 0.745).

These findings were confirmed in the matched samples. The patients with upper thoracic lesions in the S + R group had a significantly higher OS than the patients in the S group (5-year OS of 44.4% vs. 22.2% and 10-year OS of 27.8% vs. 11.1%, *χ*^2^ = 4.067, *p* = 0.044; Figure 3C). No significant difference in OS was found between the two groups of patients with middle and lower thoracic lesions (*p* = 0.889 and 0.172). The patients with upper and middle thoracic lesions in the S + R group had significantly higher DFS than the patients in the S group (upper thoracic lesions, 5-year DFS of 38.9% vs. 16.7% and 10-year DFS of 22.2% vs. 11.1%, *χ*^2^ = 4.168, *p* = 0.041, Figure 3D; middle thoracic lesions 5-year DFS of 47.1% vs. 35.3% and 10-year DFS of 32.4% vs. 8.8%, *χ*^2^ = 5.452, *p* = 0.020). No significant difference in DFS was noted between the two groups of patients with lower thoracic lesions (*p* = 0.362). (The rest of Figures were added as Supplementary Figure S2).

### Patterns of failure

**Figure 4:**
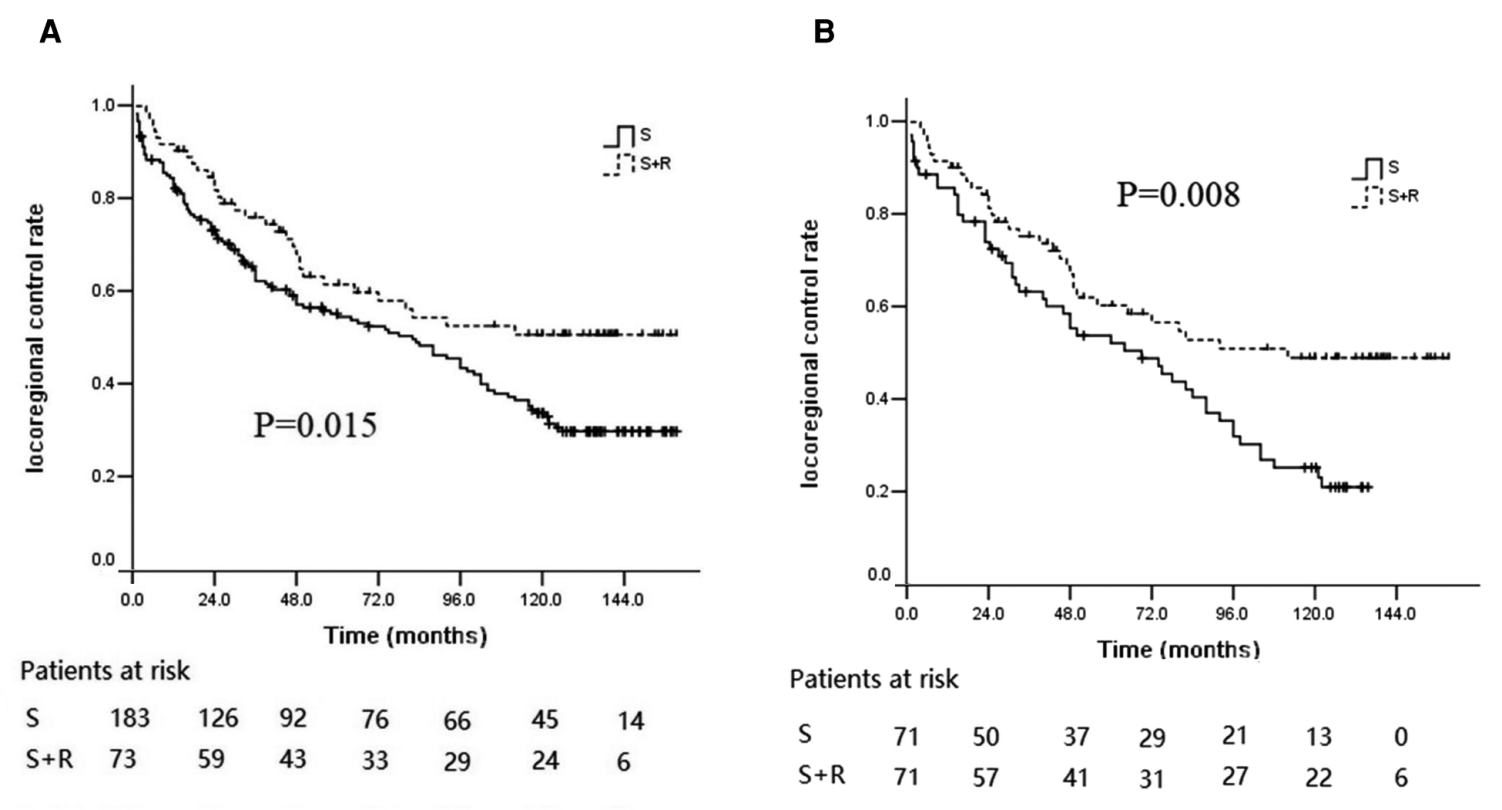
Kaplan–Meier curves of LCR between the S group and the S + R group of ESCC patients with pT3N0M0. (**A**) LCR before PSM; (**B**) LCR after PSM. LCR, locoregional control rate; S, surgery alone; S + R, surgery plus postoperative radiotherapy; PSM, propensity score matching; ESCC, Esophageal squamous cell carcinoma).

The patterns of failure identified included locoregional recurrence and distant metastasis. Treatment failure developed in 49 patients (22 with locoregional recurrence, 17 with distant metastasis, and 10 with locoregional recurrence and metastasis) in the S + R group and in 145 patients (82 with locoregional recurrence, 32 with distant metastasis, and 31 with locoregional recurrence and metastasis) in the S group. The 5- and 10-year locoregional control rates (LCRs) in the S + R group (61.5% and 50.7%) were significantly higher than the 5- and 10-year LCRs in the S group (54.50% and 33.79%, respectively) (log-rank *χ*^2^ = 5.916, *p* = 0.015; Figure 4A). After PSM, recurrence developed in 48 patients in the S + R group (22 with locoregional recurrence, 16 with distant metastasis, and 10 with locoregional recurrence and metastasis) and in 58 patients in the S group (36 with locoregional recurrence, 8 with distant metastasis, and 14 with locoregional recurrence and metastasis). The 5- and 10-year LCRs in the S + R group (60.3% and 49.0%, respectively) were significantly higher than the 5- and 10-year LCRs in the S group (52.2% and 25.3%, respectively) (log-rank *χ*^2^ = 6.997, *p* = 0.008; Figure 4B). The locoregional recurrence between the two groups were significantly different before and after PSM (*p* = 0.009 and 0.002, respectively). The patterns of failure and locoregional recurrence are summarized in Tables 2, 3. Subgroup analysis indicated that the 5- and 10-year LCRs, respectively, were 44.3% and 37.9% in patients with upper lesions; 53.0% and 31.9% in patients with middle thoracic lesions; and 72.4% and 55.2% in patients with lower thoracic lesions, (*p* = 0.006). Similarly, the 5- and 10-year LCRs, respectively, were 42.9% and 35.1% in patients with upper thoracic lesions; 42.9% and 35.1% in patients with upper thoracic lesions; 51.3% and 28.1% in patients with middle thoracic lesions; and 77.5% and 55.8% in patients with lower thoracic lesions (*p* = 0.008). The locoregional recurrence with different tumor locations are summarized in Table 4.

**Table 2:** Patterns of failure of ESCC patients with pT3N0M0.

Variables	Before PSM (*n* = 256)			After PSM (*n* = 142)		
	S + R group	S group	*p*-value	S + R group	S group	*p*-value
	*n* = 73 (%)	*n* = 183 (%)		*n* = 71 (%)	*n* = 71 (%)	
**Locoregional recurrence**	32 (43.8)	113 (61.7)	0.009	32 (45.1)	50(70.4)	0.002
Supraclavicular	7 (9.6)	13 (7.1)		7 (9.9)	9 (12.7)	
Mediastinal	19 (26.0)	62 (33.9)		19 (26.8)	23 (32.4)	
Upper abdomen	1 (1.4)	9 (4.9)		1 (1.4)	2 (2.8)	
Anastomosis	1 (1.4)	3 (1.6)		1 (1.4)	3 (4.2)	
Supraclavicular + Mediastinal	1 (1.4)	11 (6.0)		1 (1.4)	7 (9.9)	
Supraclavicular + Upper abdomen	–	3 (1.6)		–	1 (1.4)	
Mediastinal + Upper abdomen	1 (1.4)	6 (3.3)		1 (1.4)	1 (1.4)	
Mediastinal + Anastomosis	1 (1.4)	3 (1.6)		1 (1.4)	2 (2.8)	
Supraclavicular + Mediastinal + Upper abdomen	1 (1.4)	3 (1.6)		1 (1.4)	2 (2.8)	
**Distant metastasis**	27 (37.0)	63 (34.4)	0.762	26 (36.6)	22 (31.0)	0.478
Lung	7 (9.6)	25 (13.7)		7 (9.9)	7 (9.9)	
Liver	6 (8.2)	20 (10.9)		7 (9.9)	7 (9.9)	
Bone	6 (8.2)	10 (5.5)		6 (8.5)	5 (7.0)	
Brain	4 (5.5)	1 (0.5)		4 (5.6)	–	
Adrenal gland	–	2 (1.1)		–	1 (1.4)	
Lung + Liver	2 (2.7)	–		1 (1.4)	–	
Lung + Bone	1 (1.4)	3 (1.6)		1 (1.4)	1 (1.4)	
Lung + Brain	1 (1.4)	–		–	–	
Liver + Bone	–	1 (0.5)		–	–	
Brain + Bone	–	1 (0.5)		–	1 (1.4)	

ESCC, esophageal squamous cell carcinoma; PSM, propensity score matching; S, surgery alone; S + R, surgery plus postoperative radiotherapy.

**Table 3:** Locoregional recurrence of ESCC patients with pT3N0M0.

Recurrence location	Before PSM (*n* = 256)			After PSM (*n* = 142)		
	S + R group	S group	*p*-value	S + R group	S group	*p*-value
	*n* = 73 (%)	*n* = 183 (%)		*n* = 71 (%)	*n* = 71 (%)	
Locoregional recurrence	32 (43.8)	113 (61.7)	0.009	32 (45.1)	50 (70.4)	0.002
Supraclavicular	9 (12.3)	30 (16.4)	0.414	9 (12.7)	19 (26.8)	0.035
Mediastinal	23 (31.5)	85 (46.4)	0.029	23 (32.4)	37 (52.1)	0.017
Upper abdomen	3 (4.1)	21 (11.5)	0.068	3 (4.2)	6 (8.5)	0.301
Anastomosis	2 (2.7)	6 (3.3)	0.823	2 (2.8)	5 (7.0)	0.245

ESCC, esophageal squamous cell carcinoma; PSM, propensity score matching; S, surgery alone; S + R, surgery plus postoperative radiotherapy.

**Table 4:** Locoregional recurrence with different tumor location of ESCC patients with pT3N0M0.

Recurrence location	Before PSM (*n* = 256)				After PSM (*n* = 142)			
	Tumor location			*p*-value	Tumor location			*p*-value
	Upper	Middle	Lower		Upper	Middle	Lower	
	*n* = 45 (%)	*n* = 146 (%)	*n* = 65 (%)		*n* = 36 (%)	*n* = 68 (%)	*n* = 38 (%)	
Supraclavicular	17 (37.8)	21 (14.4)	1 (1.5)	<0.001	14 (38.9)	14 (20.6)	–	<0.001
Mediastinal	15 (33.3)	75 (51.4)	18 (27.7)	0.002	15 (41.7)	33 (48.5)	14 (36.8)	0.489
Upper abdomen	1 (2.2)	13 (8.9)	10 (15.4)	0.064	1 (2.8)	4 (5.9)	4 (10.5)	0.384
Anastomosis	2 (4.4)	6 (4.1)	–	0.244	2 (5.6)	5 (7.4)	–	0.249

ESCC, esophageal squamous cell carcinoma; PSM, propensity score matching; S, surgery alone; S + R, surgery plus postoperative radiotherapy.

### Toxicities

In the 73 patients accepting PORT, grade-4 and grade-5 radiation-related toxicity was not observed in any patients. Grade-3 acute toxicity was observed in 2 patients (2.7%) with esophagitis; grade-2 acute toxicity was found in 11 patients (15.1%) with esophagitis, 7 patients (9.6%) with gastritis, and 3 patients (4.1%) with pneumonitis. Late toxicity was observed in 3 patients (4.1%) with grade-3 anastomotic stenosis and in 3 patients (4.1%) with grade-2 anastomotic stenosis.

## Discussion

Surgery is still a dominant therapeutic strategy for patients with pT3N0M0 ESSC. However, the effect of single surgery remains unsatisfactory, and the recurrence rate is still high post-surgery. PORT is adopted in patients with pT3N0M0 ESCC, particularly those who are clinically under-staged before surgery, to reduce locoregional recurrence and improve survival; however, the value remains inconclusive.

In a prospective randomized study (17) of 68 ESCC patients undergoing radical resection, 33 patients received PORT and were compared to a control group of 35 patients treated with surgery alone. No significant differences in OS and DFS were noted between the two treatment groups. In the subgroup analysis of 32 patients with stage II, no significant differences were found between the irradiated (16 patients) and control groups (16 patients). The 1-, 2-, and 3-year OS rates were 80%, 48%, 35% and 87%, 53%, 38%, respectively. Another prospective randomized study (14) with a large sample size was conducted to assess the value of radiotherapy after radical resection. A total of 495 ESCC patients were randomized into the surgery-alone group consisting of 275 patients and the surgery + PORT group consisting of 220 patients. For the patients with stage pT2–3N0M0, the 1-, 3-, and 5-year OS rates were 88.2%, 56.0% and 51.3% in the surgery group and 88.6%, 64.0%, and 50.3% in the surgery+ radiotherapy group (*p* = 0.6344). The study also showed that PORT substantially reduced the incidence of recurrence in the supraclavicular and mediastinal regions (*p* < 0.001 and *p* < 0.015) but not in the upper abdomen (*p* = 0.351). Both randomized controlled studies occurred during conventional two-dimensional radiotherapy (2DRT). As everyone knows, 2DRT was always associated with more radiation-related toxicity and death resulting from radiation (23). Currently, conformal radiotherapy (cRT), including three-dimensional cRT (3D-CRT) and intensity-modulated radiotherapy (IMRT) is widely used in clinical treatment. Compared with 2DRT, cRT achieves a high target volume dose while minimizing the radiation dose to the organ at risk. Several studies have shown that compared with 2DRT, cRT improves survival and decreases toxicity in various cancers (24, 25). On the basis of the latest radiotherapy techniques and treatment planning systems, the PORT value needed to be comprehensively reevaluated in patients with pT3N0M0 ESCC.

A recent retrospective study (15) collected the clinical data of 692 patients with T3N0M0 who underwent radical resection, with or without PORT. The cohort included 344 ESCC patients with pT3N0M0 ESCC, consisting of 248 patients who underwent surgery alone and 96 patients who received surgery plus PORT. The 5-year OS of the surgery-alone and surgery-plus-PORT groups were 54.0% and 69.8%, respectively (*p* = 0.007). The 5- year recurrence-free survival of the surgery-plus-PORT group was significantly higher than that of the surgery-alone group (75.6% vs. 52.4%, *p* < 0.001). Another large-scale study (9) retrospectively analyzed 678 patients with pT3N0M0 ESCC, consisting of 583 in the surgery-alone group and 95 patients in the PORT group. The aforementioned study showed that the PORT had significant benefits for survival and could significantly reduce the incidence of locoregional recurrence. The same result was also confirmed in the matched samples after PSM. On the basis of the two aforementioned retrospective studies, radiotherapy with advanced technology can improve survival in ESCC patients with pT3N0M0. However, in a retrospective study of 249 patients with pT3N0M0 ESCC by Wang et al. (10), no significant differences in OS and DFS were found between patients treated with or without PORT. A population-based study (26) indicated that PORT was not highly associated with OS in the entire cohort of patients with pT3N0M0 EC (*p* = 0.613) or adenocarcinoma (*p* = 0.937) and squamous cell carcinoma group (*p* = 0.764). Consequently, the value of PORT for patients with pT3N0M0 ESCC remains inconclusive.

In the present retrospective study, the survival prognosis between the S + R group and the S group was not statistically different in OS (*p* = 0.810) and DFS (*p* = 0.056). The incidence of locoregional recurrence could be reduced significantly by PORT (*p* = 0.015), particularly in reducing the recurrence rate in the supraclavicular and mediastinal regions (*p* = 0.035 and 0.038), which was consistent with the result obtained by Xiao (14). Our subgroup analysis further showed that PORT was strongly associated with improved OS (*p* = 0.013) and DFS (*p* = 0.018) in patients with upper thoracic ESCC. However, PORT improved DFS (*p* = 0.018) only in patients with middle thoracic ESCC. The results were also confirmed in patients with propensity score-matched ESCC. The lymphatic drainage of the esophagus was complex, with a rich submucosal lymphatic network and a longitudinal drainage pattern, resulting in a high rate of locoregional recurrence after surgery. Some studies (9, 10, 12, 14, 27) have shown that the lymph node recurrence rates in the supraclavicular and upper mediastinal regions were considerably higher than those in the lower mediastinal or upper abdominal region after radical operation for ESCC patients. More extensive lymphadenectomy and adjuvant radiotherapy were typically used to reduce locoregional recurrence and subsequently improve survival after surgery (15). However, the routine operation for thoracic EC is two-field esophagectomy; lymphadenectomy is seldom used for the cervical region. Lymph nodes in the upper mediastinum (specifically above the aortic arch) are usually dissected incompletely, resulting in the potential metastasis of lymph nodes in the cervical region and the upper mediastinum during surgery. By contrast, the lower mediastinum and the upper abdominal regions can be well exposed, allowing a more thorough dissection of the lymph nodes. Consequently, metastasis of lymph nodes in the supraclavicular and upper mediastina commonly occurs in patients with ESCC. There was obvious advantage for upper mediastinal lymph node dissection in 3-field lymphadenectomy compared with the 2-field lymphadenectomy. A recent meta-analysis (28) showed that 3-field lymphadenectomy comparing with 2-field lymphadenectomy seemed associated with improved 5-year overall survival. However, another meta-analysis (29) indicated that there were no significant differences between 2-field and 3-field lymphadenectomy group in OS and the number of positive lymph nodes, although more lymph nodes would be detected and obtained from 3-field lymphadenectomy. No statistical differences in cervical nodal recurrence, anastomotic stenosis and recurrent laryngeal nerve injury were observed. The recurrence pattern after radical surgery of upper thoracic esophageal cancer showed markedly high recurrence rates in the upper mediastinum and supraclavicular regions (30). Similarly, the patterns of failure in the current study determined that the supraclavicular and mediastinal regions were the most common sites of recurrence, and lymph node recurrence in the supraclavicular region was much higher in ESCC patients with upper thoracic lesions. With the aforementioned factors considered, surgery of the upper-thoracic segment of the esophagus entails greater difficulty, compared with that of the middle or lower thoracic segment. This difference could be associated with more locoregional recurrence, leading to a decline in survival. PORT in the supraclavicular region and the mediastinum could kill potentially metastatic tumor cells. Thus, PORT could be beneficial to the survival of patients with upper thoracic esophageal cancer.

This study has several limitations. Firstly, this research is a retrospective study. However, we simulated randomization by propensity score matching to eliminate potential bias by creating two comparable groups. Secondly, it was a single center study. However, the inclusion criteria ensured homogeneity of treatment with a single stage to enhance the reliability of the results. Thirdly, the small scale of patients in some subgroups may limit statistical power.

## Conclusion

In conclusion, the combination of surgery and postoperative radiotherapy is superior in local control to surgery-alone in pT3N0M0-stage ESCC. Postoperative radiotherapy may be associated with a survival benefit for patients with pT3N0M0 upper thoracic ESCC. A multicenter, randomized phase III clinical trial is required to confirm the results of this study.

## Data Availability

The original contributions presented in the study are included in the article/Supplementary Material, further inquiries can be directed to the corresponding author/s.

## References

[1] Bray F Ferlay J Soerjomataram I Siegel R Torre L Jemal A. Global cancer statistics 2018: gLOBOCAN estimates of incidence and mortality worldwide for 36 cancers in 185 countries. CA Cancer J Clin. (2018) 68:394–424. 10.3322/caac.21492 30207593

[2] Feng RM Zong YN Cao SM Xu RH. Current cancer situation in China: good or bad news from the 2018 global cancer statistics? Cancer Commun. (2019) 39(1):1–12. 10.1186/s40880-018-0346-4 PMC6487510 31030667

[3] Chitti B Pham A Marcott S Wang X Potters L Wernicke A Temporal changes in esophageal cancer mortality by geographic region: a population-based analysis. Cureus. (2018) 10(11):e3596. 10.7759/cureus.3596 30680257 PMC6338397

[4] Enziger PC Mayer RJ. Esophageal cancer. N Eng J Med. (2003) 349(23):2241–52. 10.1056/NEJMra035010 14657432

[5] Eyck BM van Lanschot JJB Hulshof MCCM van der Wilk BJ Shapiro J van Hagen P Outcome of patients treated within and outside a randomized clinical trial on neoadjuvant chemoradiotherapy plus surgery for esophageal cancer: extrapolation of a randomized clinical trial (CROSS). J Clin Oncol. (2021) 39(18):1995–2004. 10.1200/JCO.20.03614 33891478

[6] Mao Y S Gao S G Wang Q Shi XT Li Y Gao WW Epidemiological characteristic and current status of surgical treatment for esophageal cancer by analysis of national registry database. Chin J Oncol. (2020) 42(3):228–33. 10.3760/cma.j.cn112152-20191112-00729 32252202

[7] Zeng Y Yu W Liu Q Yu WW Zhu ZF Zhao WX Difference in failure patterns of pT3-4N0-3M0 esophageal cancer treated by surgery vs surgery plus radiotherapy. World J Gastrointest Oncol. (2019) 11(12):1172–81. 10.4251/wjgo.v11.i12.1172 31908722 PMC6937439

[8] Liu T Liu W Zhang H Ren C Chen J Dang J The role of postoperative radiotherapy for radically resected esophageal squamous cell carcinoma: a systemic review and meta-analysis. J Thorac Dis. (2018) 10(7):4403–12. 10.21037/jtd.2018.06.65 30174889 PMC6105941

[9] Yang JS Zhang WC Xiao ZF Wang Q Zhou Z Zhang H The impact of postoperative conformal radiotherapy after radical surgery on survival and recurrence in pathologic T3N0M0 esophageal carcinoma: a propensity score-matched analysis. J Thorac Oncol. (2017) 12(7):1143–51. 10.1016/j.jtho.2017.03.024 28411098

[10] Wang Y Wang L Yang Q Li j Qi Z He M Factors on prognosis in patients of stage pT3N0M0 thoracic esophageal squamous cell carcinoma after two field esophagectomy. J Cancer Res Ther. (2015) 11(Suppl 1):C16–23. 10.4103/0973-1482.163833 26323918

[11] Wang Q Peng L Li T Dai W Jiang Y Xie T Postoperative chemotherapy for thoracic pathological T3N0M0 esophageal squamous cell carcinoma. Ann Surg Oncol. (2020) 27(5):1488–95. 10.1245/s10434-019-08112-1 31974708

[12] Zhang X Yang Y Sun Y Ye B Guo X Mao T Adjuvant therapy for pathological T3N0M0 esophageal squamous cell carcinoma. J Thorac Dis. (2019) 11(6):2512–22. 10.21037/jtd.2019.05.70 31372288 PMC6626811

[13] Gu X Ge Y Liu J Ding Q Chu J Tian G Impact of chemotherapy on prognosis of resectable pathological T3N0M0 esophageal cancer patients: a population-based study. Future Oncol. (2021) 17(30):3925–40. 10.2217/fon-2020-1084 34291648

[14] Xiao ZF Yang ZY Liang J Miao YJ Wang M Yin WB Value of radiotherapy after radical surgery for esophageal carcinoma: a report of 495 patients. Ann Thorac Surg. (2003) 75(2):331–6. 10.1016/S0003-4975(02)04401-6 12607634

[15] Chen SB Weng HR Wang G Liu DT Li H Zhang H The impact of adjuvant radiotherapy on radically resected T3 esophageal squamous cell carcinoma. J Cancer Res Clin Oncol. (2016) 142(1):277–86. 10.1007/s00432-015-2041-z 26328915 PMC11819054

[16] Chen G Wang Z Liu XY Zhang MY Liu FY. Clinical study of modified ivor-lewis esophagectomy plus adjuvant radiotherapy for local control of stage IIA squamous cell carcinoma in the mid-thoracic esophagus. Eur J Cardiothorac Surg. (2009) 35(1):1–7. 10.1016/j.ejcts.2008.09.002 18926712

[17] Zieren HU Müller JM Jacobi CA Pichlmaier H Müller RP Staar S. Adjuvant postoperative radiation therapy after curative resection of squamous cell carcinoma of the thoracic esophagus: a prospective randomized study. World J Surg. (1995) 19(3):444–9. 10.1007/BF00299187 7639004

[18] Chen J Zhu J Pan J Zhu K Zheng X Chen M Postoperative radiotherapy improved survival of poor prognostic squamous cell carcinoma esophagus. Ann Thorac Surg. (2010) 90(2):435–42. 10.1016/j.athoracsur.2010.04.002 20667325

[19] Worni M Martin J Gloor B Pietrobon R D'Amico TA Akushevich I Does surgery improve outcomes for esophageal squamous cell carcinoma? An analysis using the surveillance epidemiology and end results registry from 1998 to 2008. J Am Coll Surg. (2012) 215(5):643–51. 10.1016/j.jamcollsurg.2012.07.006 23084493 PMC3479433

[20] Zhang W Liu X Xiao Z Zhang H Chen D Feng Q Postoperative intensity modulated radiotherapy improved survival in lymph node-positive or stage III thoracic esophageal squamous cell carcinoma. Oncol Res Treat. (2015) 38(3):97–102. 10.1159/000375391 25792080

[21] Schreiber D Rineer J Vongtama D Wortham A Han P Schwartz D Impact of postoperative radiation after esophagectomy for esophageal cancer. J Thorac Oncol. (2010) 5(2):244–50. 10.1097/JTO.0b013e3181c5e34f 20009774

[22] Shridhar R Weber J Hoffe SE Almhanna K Karl R Meredith K. Adjuvant radiation therapy and lymphadenectomy in esophageal cancer: a SEER database analysis. J Gastrointest Surg. (2013) 17(8):1339–45. 10.1007/s11605-013-2192-7 23749498

[23] Fok M Sham JS Choy D Cheng SW Wong J. Postoperative radiotherapy for carcinoma of the esophagus: a prospective, randomized controlled study. Surgery. (1993) 113(2):138–47. 10.1063/1.2789709 8430362

[24] Sher DJ Koshy M Liptay MJ Fidler MJ. Influence of conformal radiotherapy technique on survival after chemoradiotherapy for patients with stage III non-small cell lung cancer in the national cancer data base. Cancer. (2014) 120(13):2060–8. 10.1002/cncr.28677 24692108

[25] Marta GN Silva V de Andrade Carvalho H de Arruda FF Hanna SA Gadia R Intensity-modulated radiation therapy for head and neck cancer: systematic review and meta-analysis. Radiother Oncol. (2014) 110(1):9–15. 10.1016/j.radonc.2013.11.010 24332675

[26] Ge Y Yin L Tan M Dai W Jiang Y Chen L Impact of postoperative radiotherapy for T3N0M0 esophageal cancer patients: a population-based study. Clin Transl Med. (2020) 10(3):e143. 10.1002/ctm2.143 32722868 PMC7418799

[27] Li CL Zhang FL Wang YD Han C Sun GG Liu Q Characteristics of recurrence after radical esophagectomy with two-field lymph node dissection for thoracic esophageal cancer. Oncol Lett. (2013) 5(1):355–9. 10.3892/ol.2012.946 23255948 PMC3525472

[28] Bona D Lombardo F Matsushima K Cavalli M Lastraioli C Bonitta G Three-field versus two-field lymphadenectomy for esophageal squamous cell carcinoma: a long-term survival meta-analysis. SURGERY. (2022) 171(4):940–7. 10.1016/j.surg.2021.08.029 34544603

[29] Wang J Yang Y Shaik S Three-Field versus two-field lymphadenectomy for esophageal squamous cell carcinoma: a meta-analysis. J SURG RES. (2020) 255(11):195–204. 10.1016/j.jss.2020.05.057 32563760

[30] Wang Y Zhang L Ye D Xia W Jiang J Wang X A retrospective study of pattern of recurrence after radical surgery for thoracic esophageal carcinoma with or without postoperative radiotherapy. Oncol Lett. (2018) 15(3):4033–9. 10.3892/ol.2018.7807 29556283 PMC5843998

