# Supplementary material for: Patterns of failure and long-term outcome of postoperative radiotherapy on the survival of patients with pathological T3N0M0 esophageal cancer
Source: Front Surg. 2022 Sep 2;9:959568. doi: 10.3389/fsurg.2022.959568 (PMC9479334; doi:10.3389/fsurg.2022.959568)
Supplement: Supplementary file 1 [file Table_5.docx]

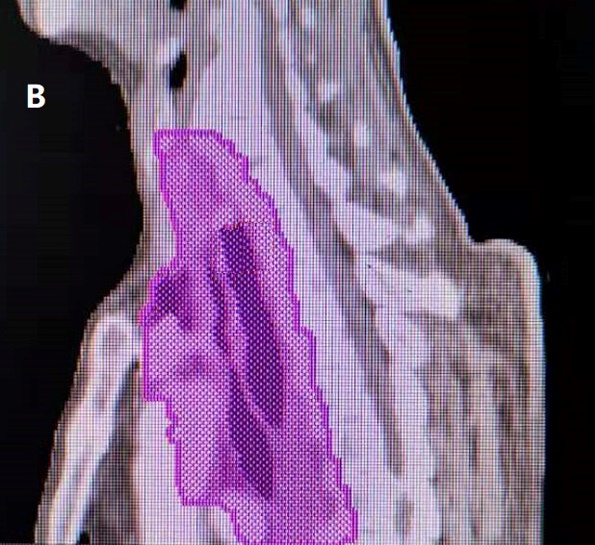

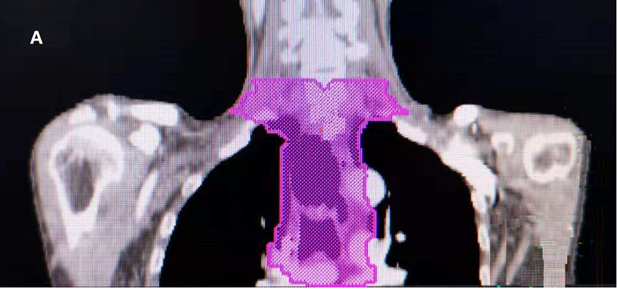


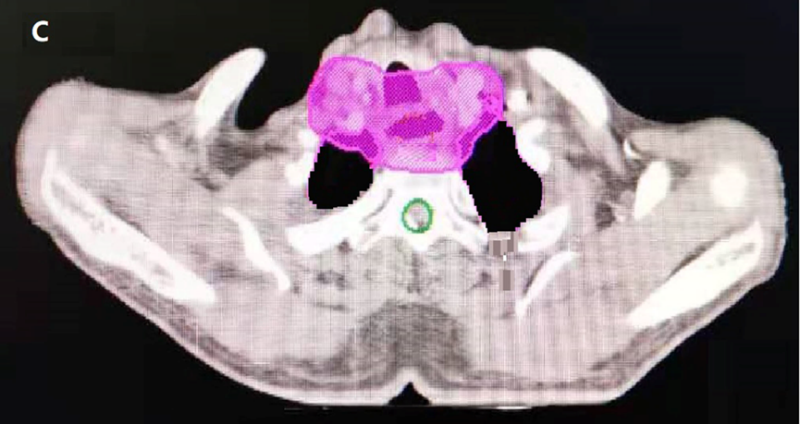


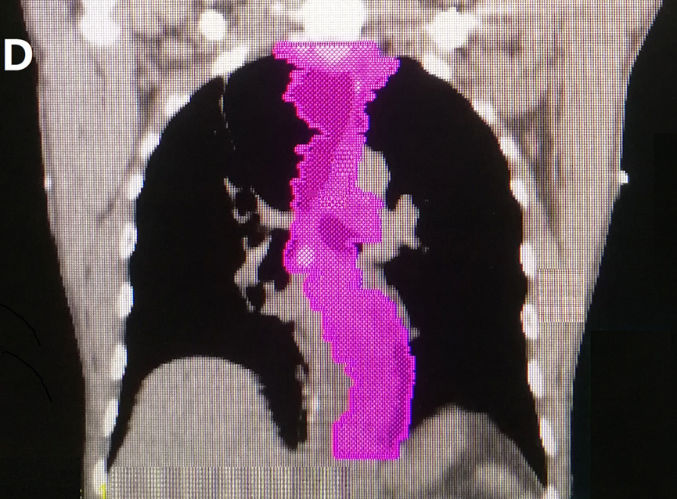


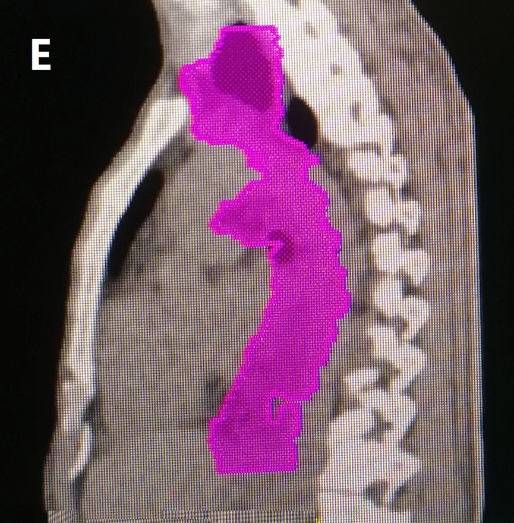


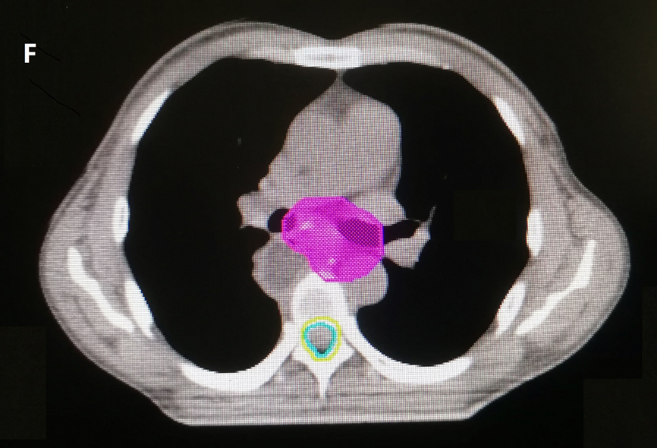


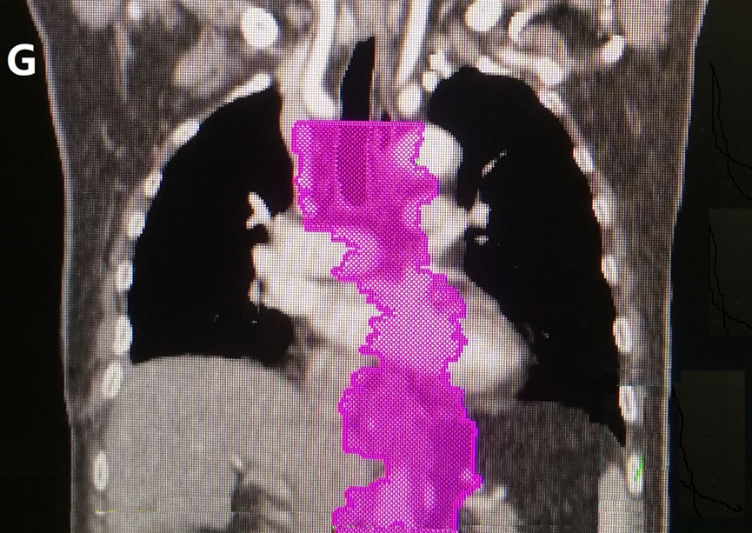


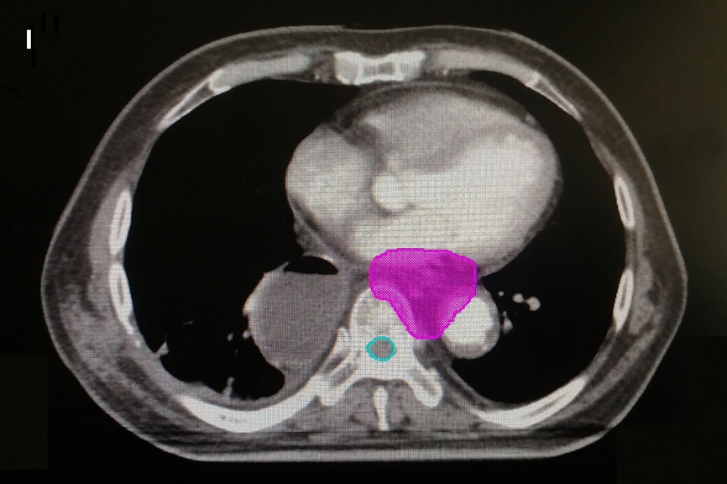

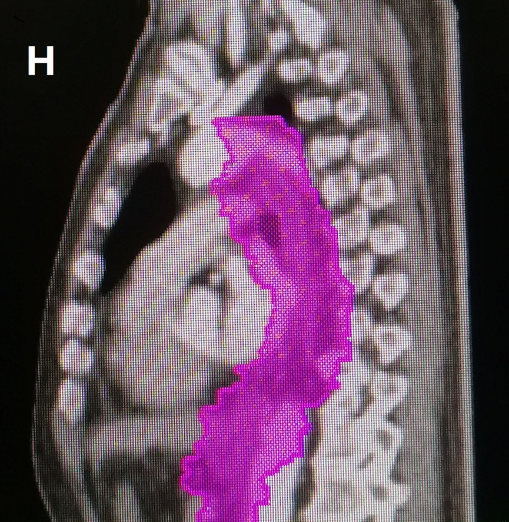


supplementary Figure 1. The target delineation pictures of CTV with different tumor site. A, B and C, the axial, sagittal and coronal of up thoracic lesions, D, E and F, the axial, sagittal and coronal of middle thoracic lesions, G, H and I, the axial, sagittal and coronal of lower thoracic lesions, respectively.
