# Supplementary material for: Patterns of failure and long-term outcome of postoperative radiotherapy on the survival of patients with pathological T3N0M0 esophageal cancer
Source: Front Surg. 2022 Sep 2;9:959568. doi: 10.3389/fsurg.2022.959568 (PMC9479334; doi:10.3389/fsurg.2022.959568)
Supplement: Supplementary file 2 [file Data_Sheet_1.zip › Supplementary Figure 2/补充图片.docx]

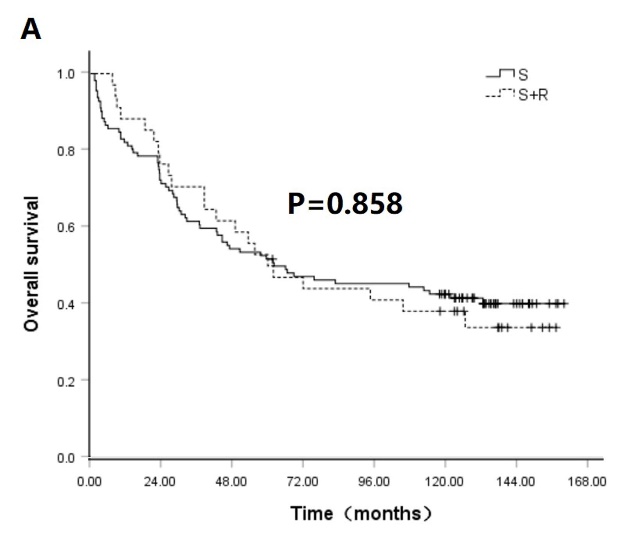


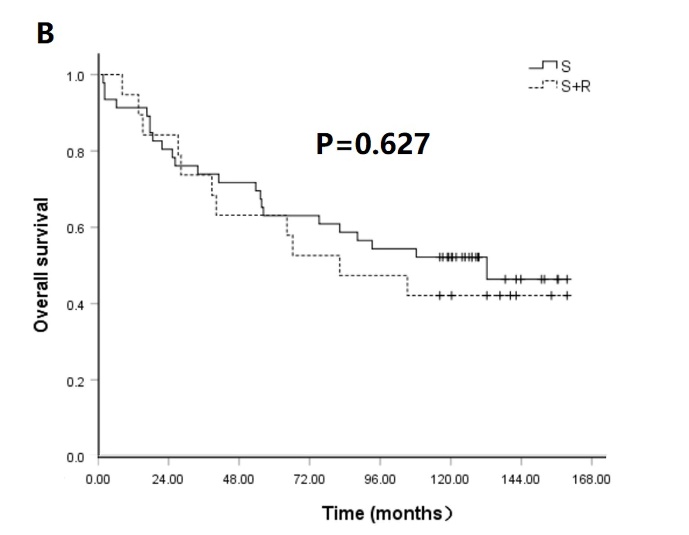


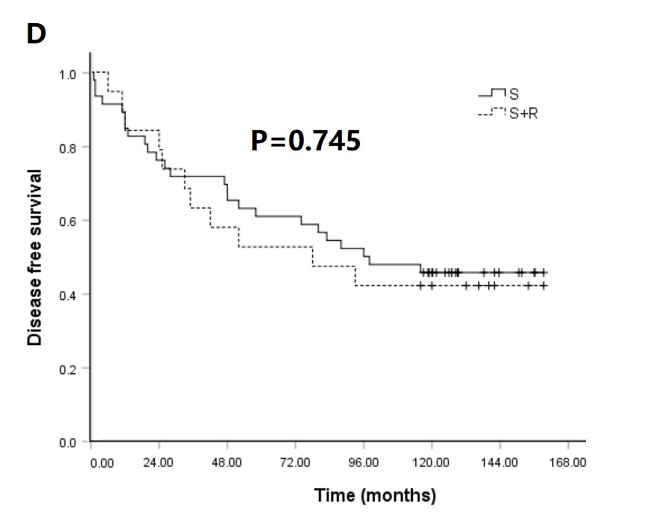

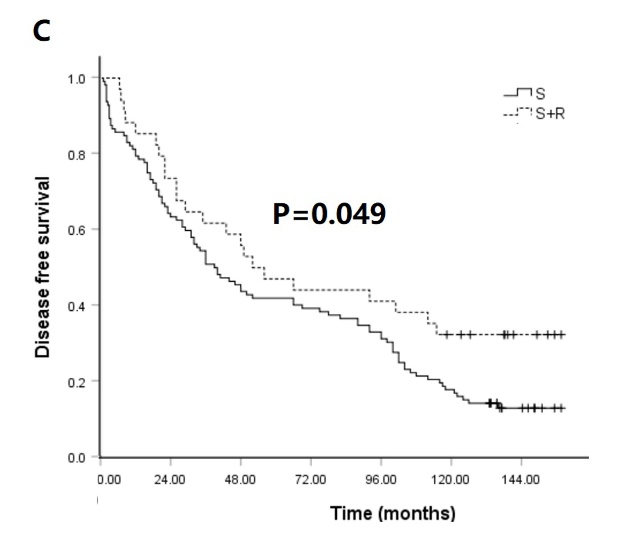


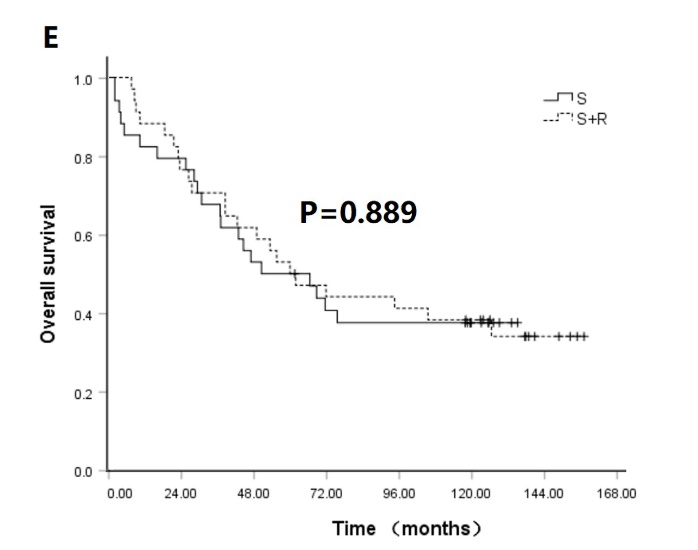


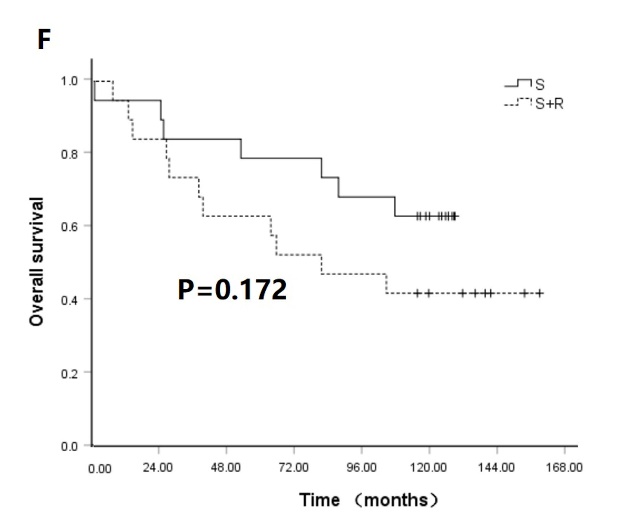


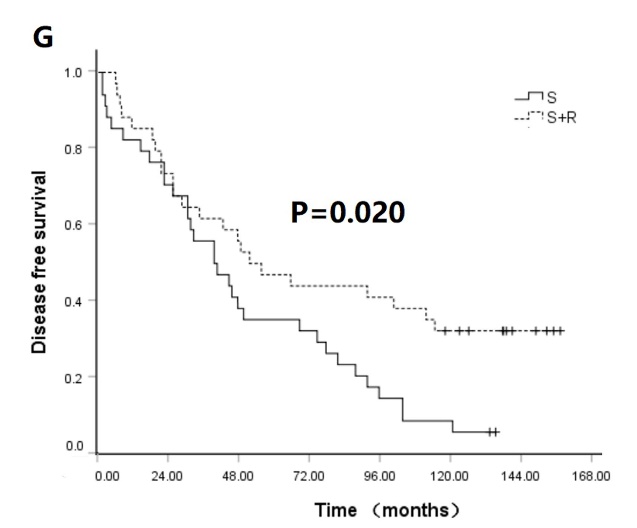

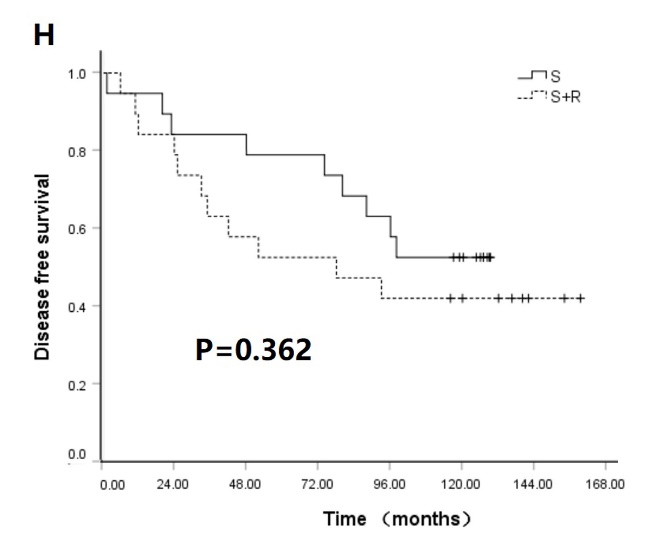


Supplementary Figure 2. Kaplan–Meier analysis of survival between the S group and the S+R group of ESCC patients with pT3N0M0 in different tumor locations before and after PSM.

(Before PSM: A and B, OS of middle and lower thoracic lesions, respectively; C and D, DFS of middle and lower thoracic lesions, respectively; After PSM: E and F, OS of middle and lower thoracic lesions, respectively; G and H, DFS of middle and lower thoracic lesions, respectively. PSM, propensity score matching; S, surgery alone; S+R, surgery plus postoperative radiotherapy; ESCC, Esophageal squamous cell carcinoma; OS, overall survival; DFS, disease free survival.)
