# Supplementary figures and images for: Patterns of failure and long-term outcome of postoperative radiotherapy on the survival of patients with pathological T3N0M0 esophageal cancer
Source: Front Surg. 2022 Sep 2;9:959568. doi: 10.3389/fsurg.2022.959568 (PMC9479334; doi:10.3389/fsurg.2022.959568)

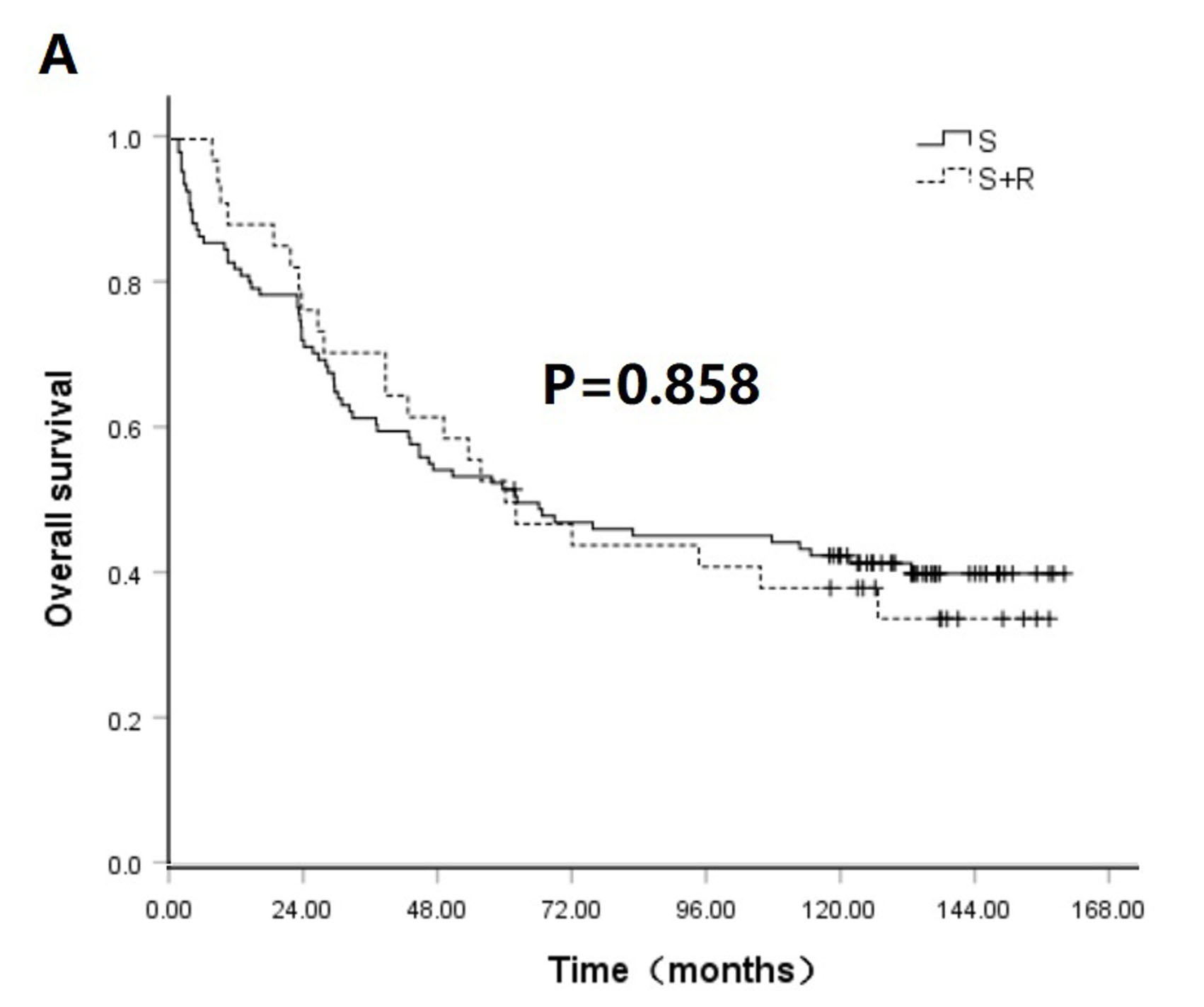

Supplement: Supplementary file 2 [file Data_Sheet_1.zip › Supplementary Figure 2/2A.jpg]

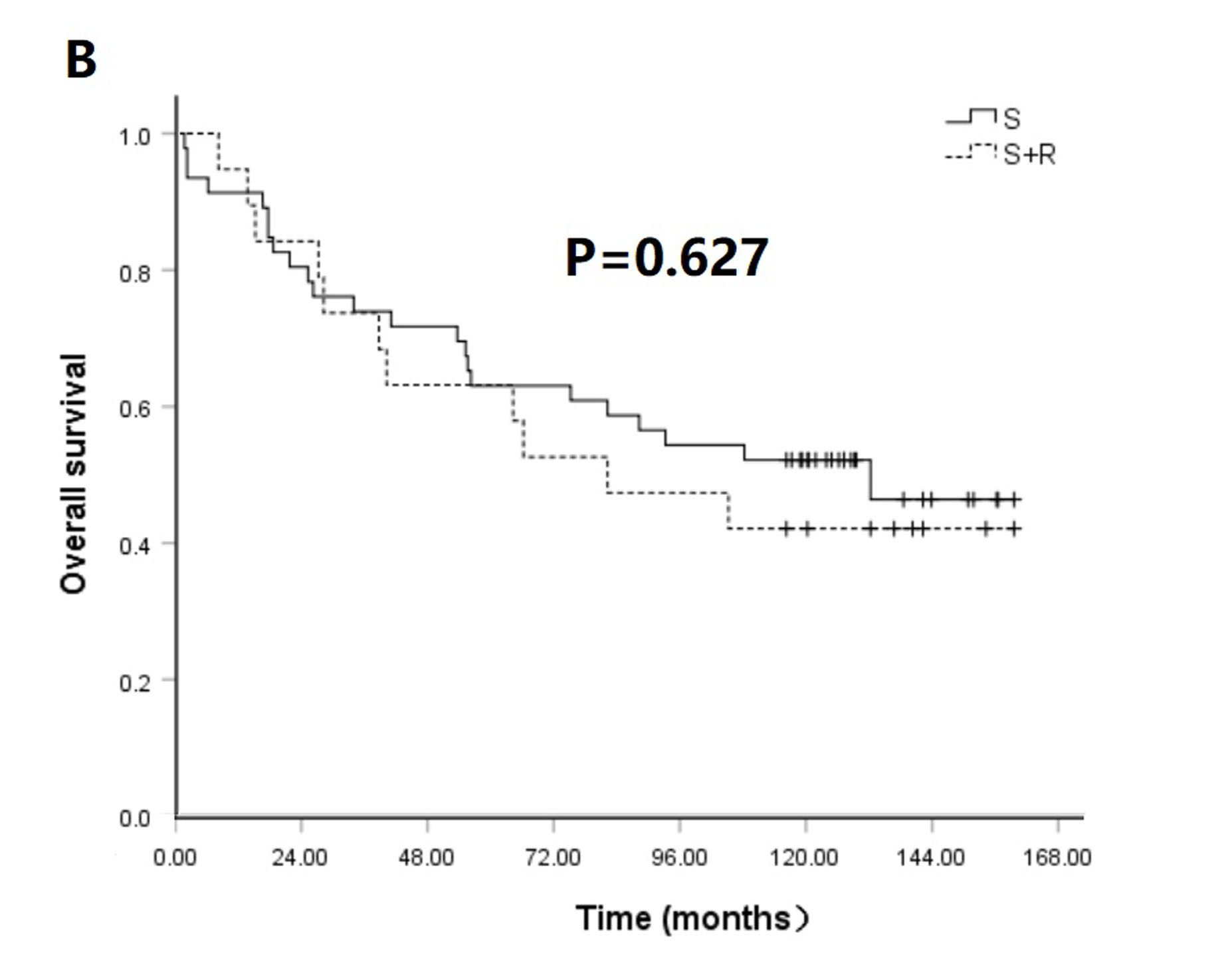

Supplement: Supplementary file 2 [file Data_Sheet_1.zip › Supplementary Figure 2/2B.jpg]

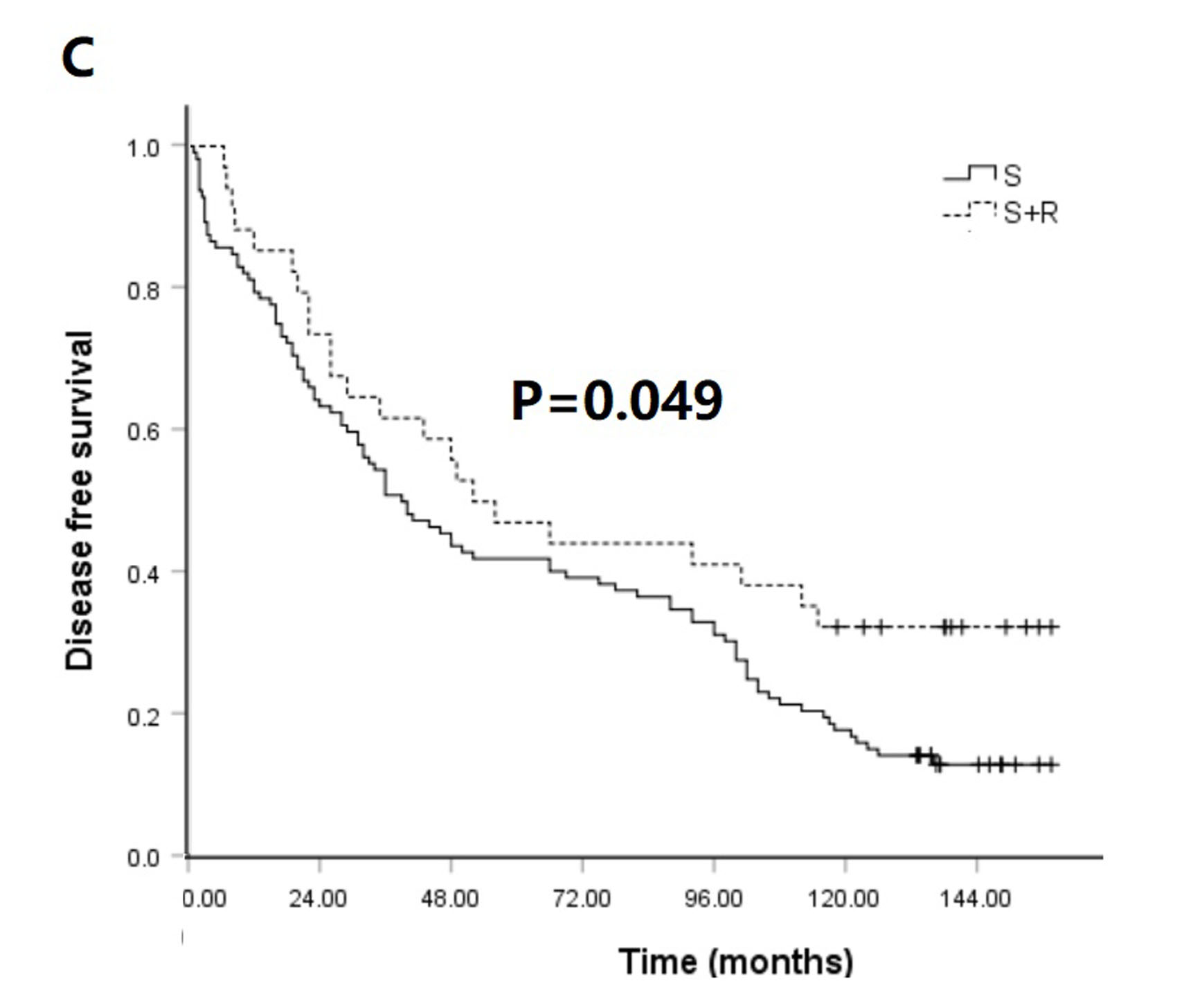

Supplement: Supplementary file 2 [file Data_Sheet_1.zip › Supplementary Figure 2/2C.jpg]

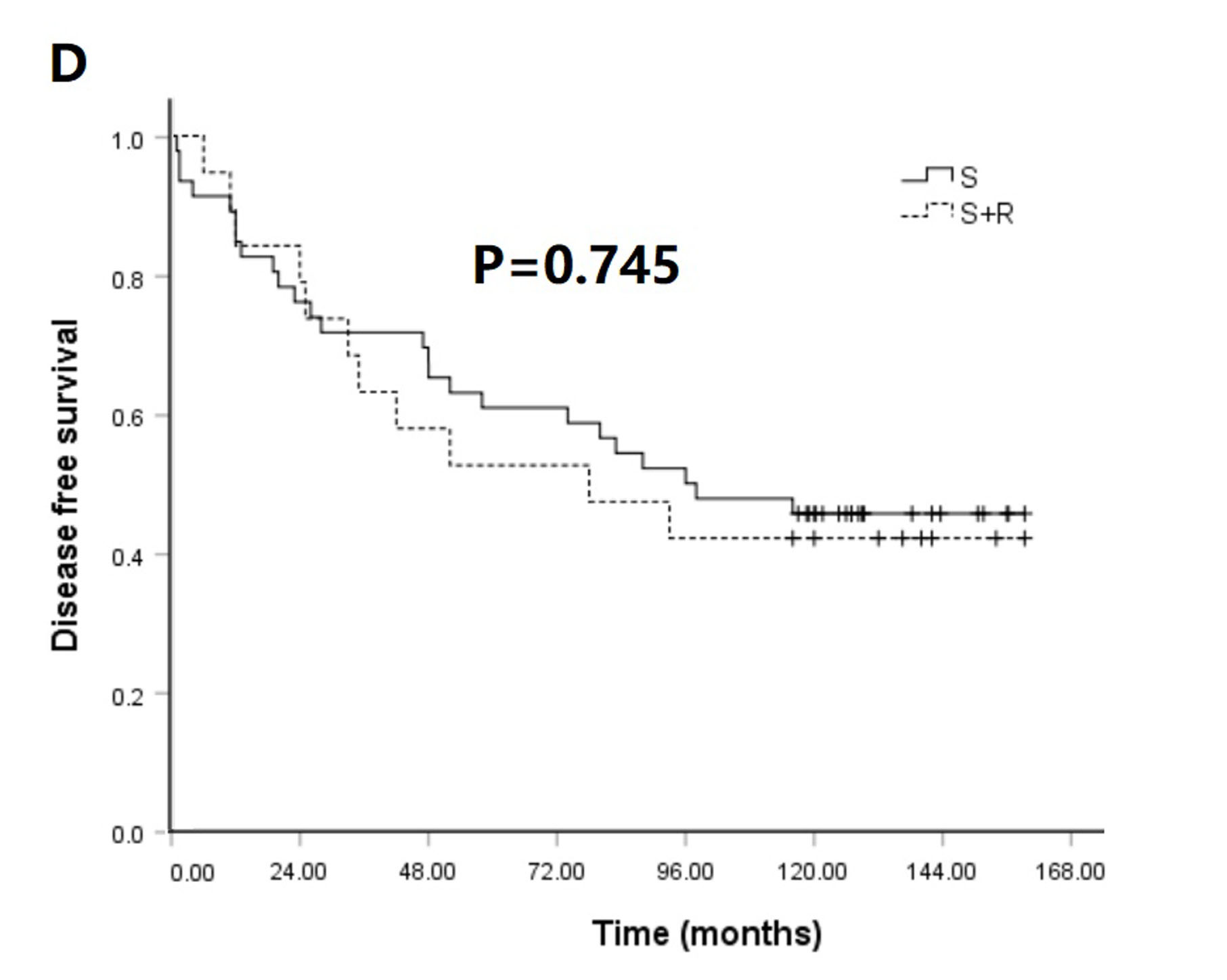

Supplement: Supplementary file 2 [file Data_Sheet_1.zip › Supplementary Figure 2/2D.jpg]

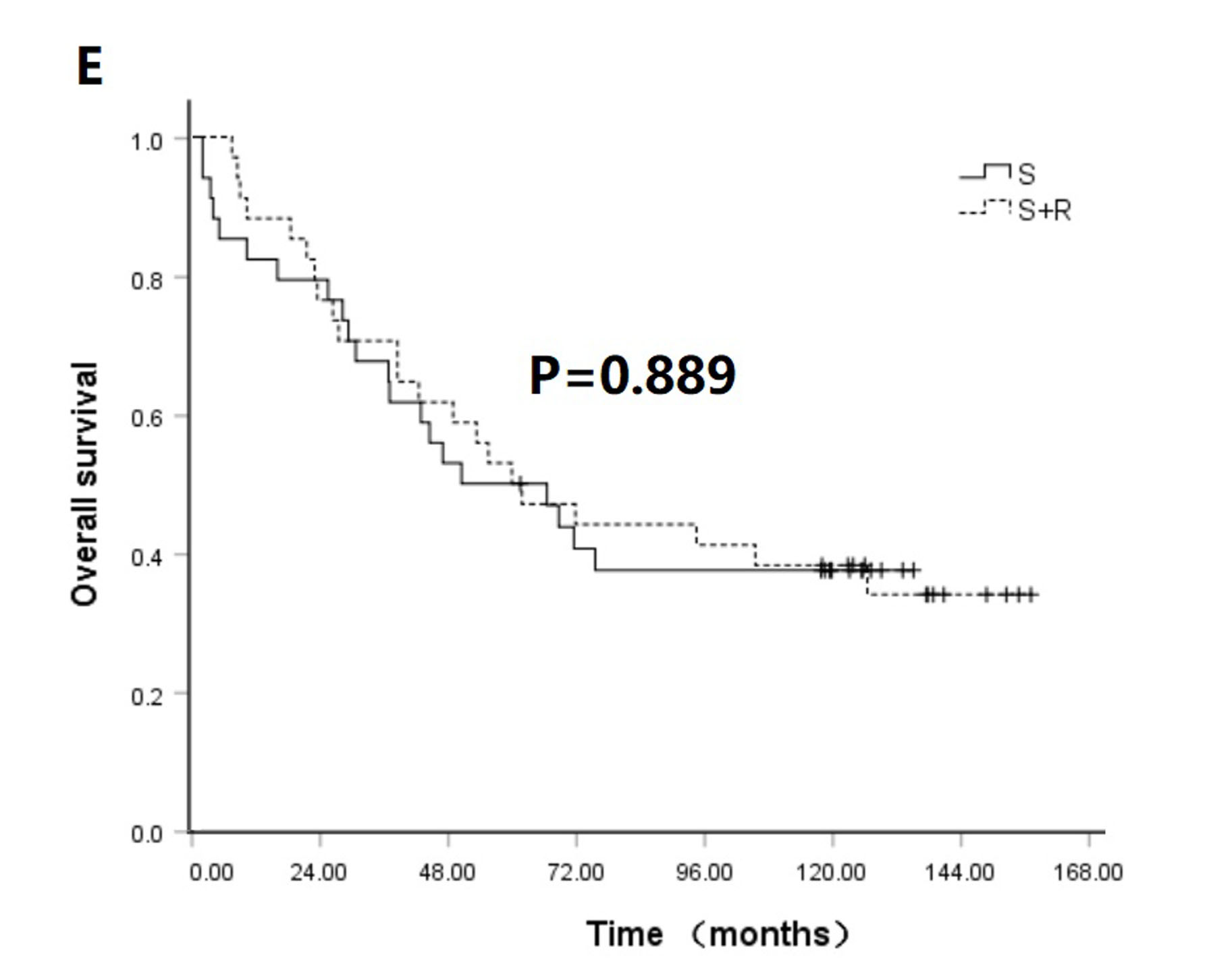

Supplement: Supplementary file 2 [file Data_Sheet_1.zip › Supplementary Figure 2/2E.jpg]

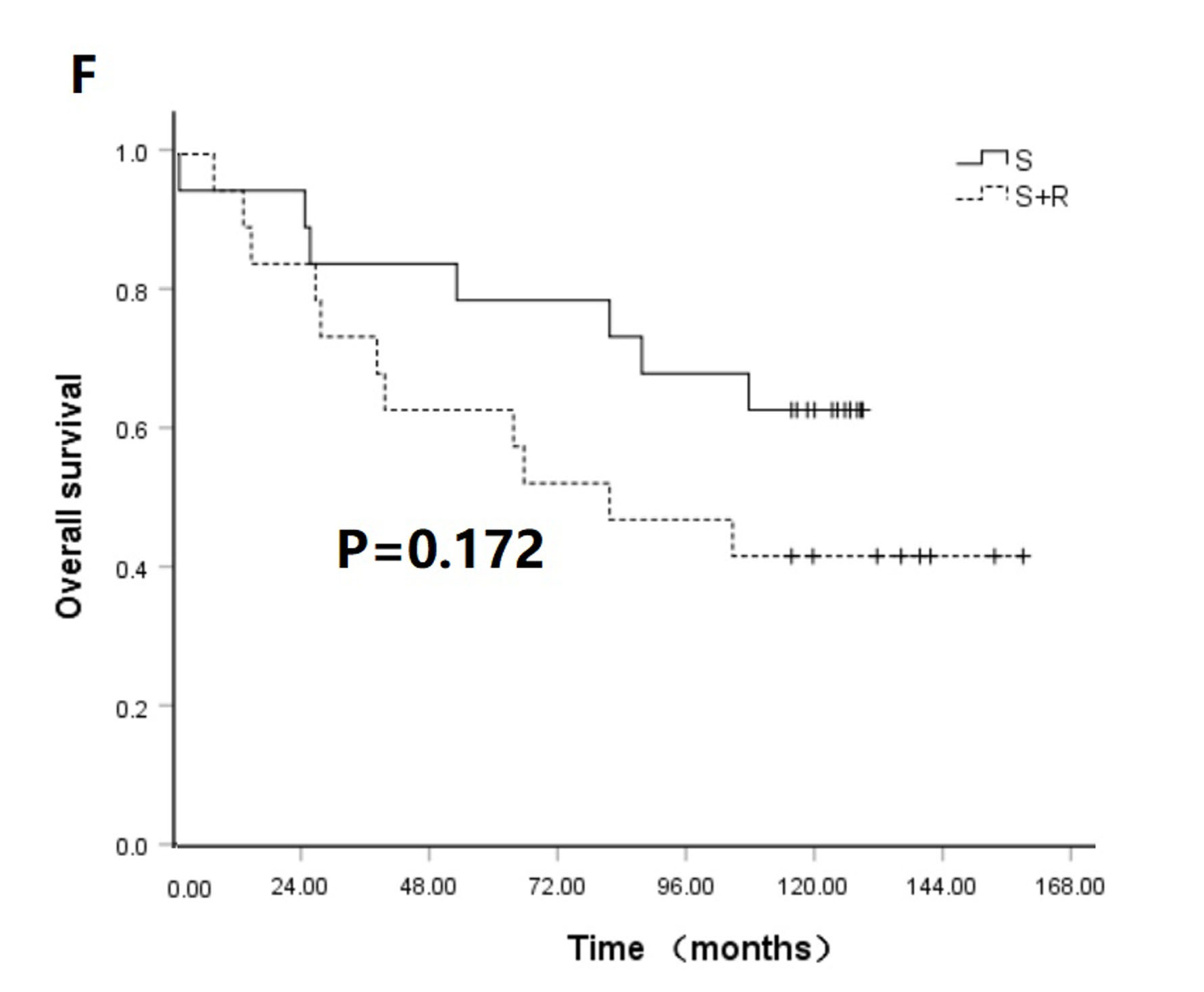

Supplement: Supplementary file 2 [file Data_Sheet_1.zip › Supplementary Figure 2/2F.jpg]

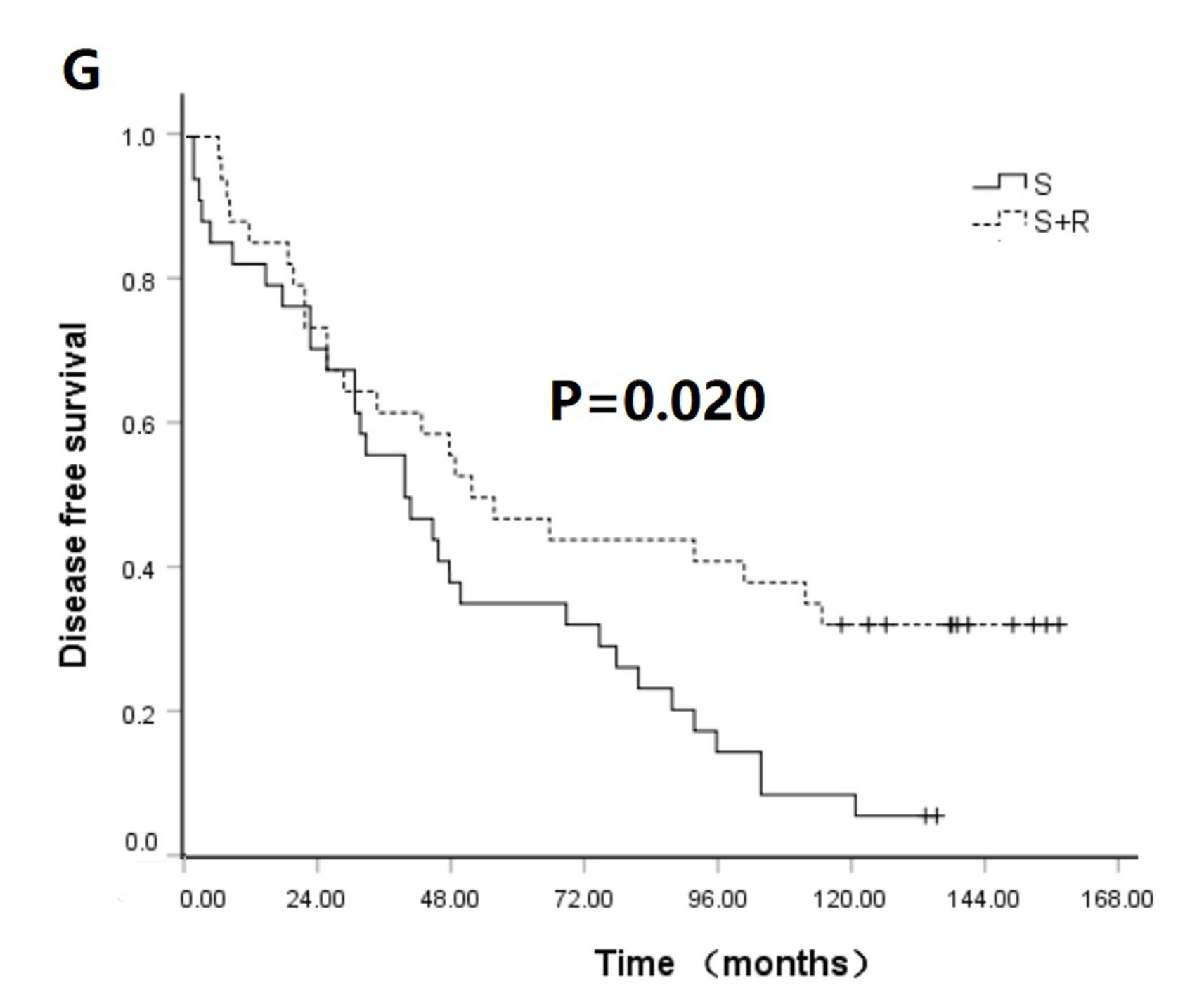

Supplement: Supplementary file 2 [file Data_Sheet_1.zip › Supplementary Figure 2/2G.jpg]

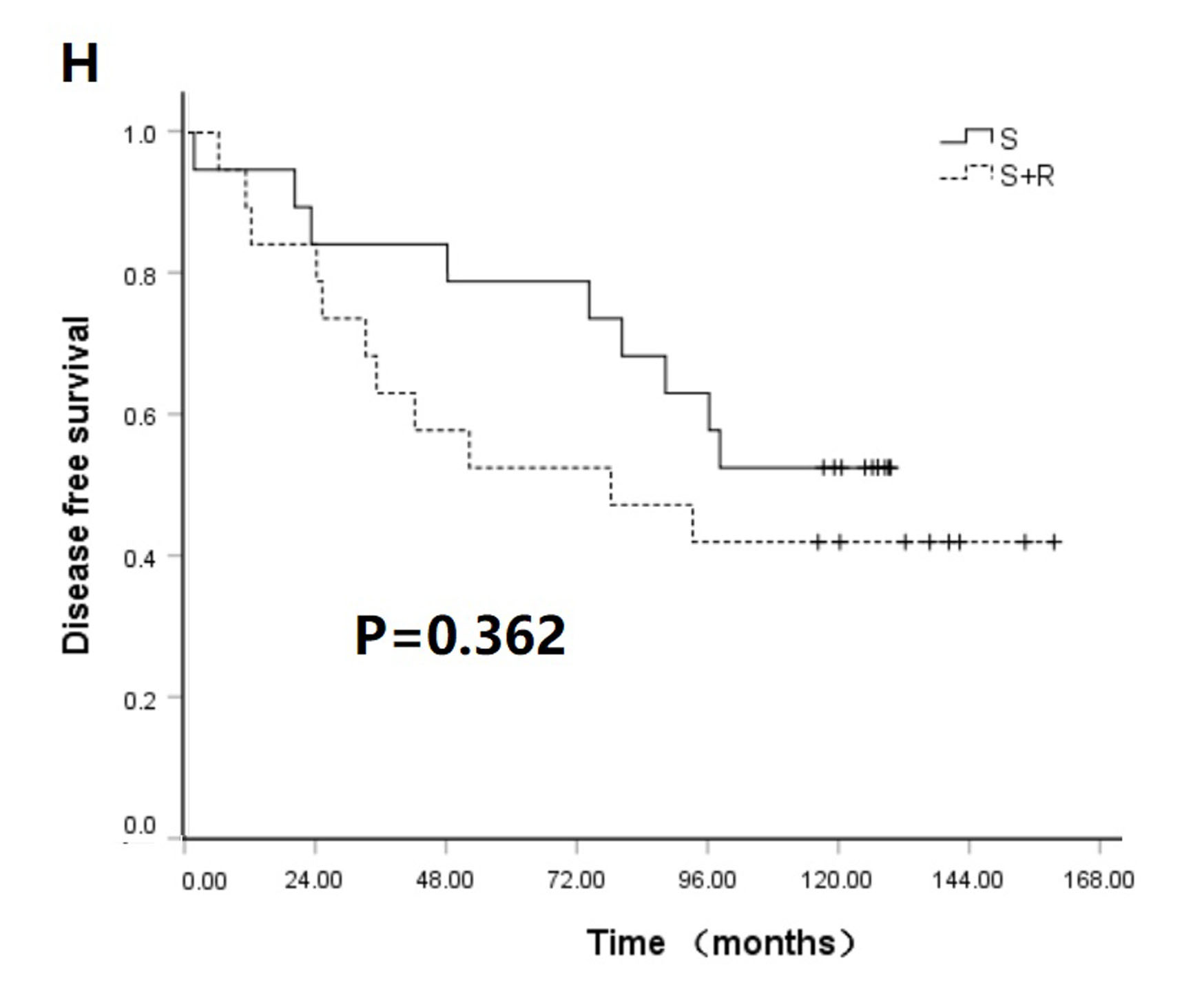

Supplement: Supplementary file 2 [file Data_Sheet_1.zip › Supplementary Figure 2/2H.jpg]
